# Transcriptome analyses revealed the ultraviolet B irradiation and phytohormone gibberellins coordinately promoted the accumulation of artemisinin in *Artemisia annua* L.

**DOI:** 10.1186/s13020-020-00344-8

**Published:** 2020-07-01

**Authors:** Tingyu Ma, Han Gao, Dong Zhang, Yuhua Shi, Tianyuan Zhang, Xiaofeng Shen, Lan Wu, Li Xiang, Shilin Chen

**Affiliations:** 1 Key Laboratory of Beijing for Identification and Safety Evaluation of Chinese Medicine, Institute of Chinese Materia Medica, China Academy of Chinese Medical Sciences, Beijing, 100700 China; 2 School of Chemistry, Chemical Engineering and Life Science, Wuhan University of Technology, Wuhan, 430070 China; 3 State Key Laboratory of Agricultural Microbiology, Huazhong Agricultural University, Wuhan, 430070 China

**Keywords:** *Artemisia annua*, Artemisinin biosynthesis, Ultraviolet B irradiation (UV-B), Gibberellins (GAs), RNA sequencing (RNA-seq), Gene co-expression

## Abstract

**Background:**

Artemisinin-based combination therapy has become the preferred approach for treating malaria and has successfully reduced malaria-related mortality. Currently, the main source of artemisinin is *Artemisia annua* L., and thus, it is of strategic importance to enhance artemisinin contents in *A. annua* plants. Phytohormones and illumination are known to be important external environmental factor that can have notable effects on the production of secondary metabolite. The activities of different hormones can be influenced to varying degrees by light, and thus light and hormones may jointly regulate various processes in plants. Here, we performed transcriptome and metabolome analyses revealed that ultraviolet B irradiation and phytohormone gibberellins coordinately promoted the accumulation of artemisinin in *Artemisia annua*.

**Methods:**

Artemisinin analysis was performed by ultra-high performance liquid chromatography-tandem quadrupole mass spectrometry (UPLC-ESI-QqQ-MS/MS). RNA sequencing, GO and KEGG enrichment analysis were applied to analyzing the differentially expressed genes (DEGs) under ultraviolet B irradiation and gibberellins treatments. Weighted gene co-expression network (WGCNA) analyzed the genes in artemisinin‑related modules and identified candidate hub genes in these modules.

**Results:**

In this study, we found that cross-talk between UV-B and GA induced processes leading to modifications in artemisinin accumulation. A total of 14,762 genes differentially expressed (DEGs) among different treatments were identified by transcriptome analysis. UV-B and GA treatments enhanced the accumulation of artemisinin by up-regulating the expression of the key artemisinin biosynthesis genes ADS and CYP71AV1. According to the high degree value and high expression level, a total of 84 co-expressed transcription factors were identified. Among them, MYB and NAC TFs mainly involved in regulating the biosynthesis of artemisinin. Weighted gene co-expression network analysis revealed that GA + UV in blue modules was positively correlated with artemisinin synthesis, suggesting that the candidate hub genes in these modules should be up-regulated to enhance artemisinin synthesis in response to GA + UV treatment.

**Conclusion:**

Our study demonstrated the co-regulation of artemisinin biosynthetic pathway genes under ultraviolet B irradiation and phytohormone gibberellins treatment. The co-expression was analysis revealed that the selected MYB and NAC TFs might have regulated the artemisinin biosynthesis gene expression with ADS and CYP71AV1 genes. Weighted gene co-expression network analysis revealed that GA + UV treatment in blue modules was positively correlated with artemisinin synthesis. We established the network to distinguish candidate hub genes in blue modules might be up-regulated to enhance artemisinin synthesis in response to GA + UV treatment.

## Background

Malaria is one of the three most life-threatening diseases in the world. Currently, artemisinin is used as a first line and effective drug for the treatment of malaria, which is caused by the Plasmodium falciparum parasite [1]. The WHO recommended the use of artemisinin-based combination therapies (ACTs), which is the most effective malaria treatment available at present [2]. The sesquiterpene artemisinin derived from the medicinal plant *Artemisia annua*, which is remain the main and only natural source of artemisinin. The dry weight yield of 1.5% artemisinin was obtained by breeding *Artemisia annua* [3]. Although artemisinin has been chemically synthesized, this method has complex steps and thus is far from industrialization, as is the semi-synthetic of artemisinin by bioengineering [4, 5]. Consequently, enhancing the content of artemisinin in *A. annua* plants would be highly desirable.

Recent publications have reported that numerous factors have been reported to regulate the biosynthesis of artemisinin, including light, plant hormones, temperature, and saline and drought stresses [6–10]. Illumination is a key external environmental factor that can have notable effects on the production of secondary metabolites [11, 12]. According to the length of wavelength, light can be divided into far-red, red, blue, and ultraviolet light. Ultraviolet light can be divided into UV-A, UV-B and UV-C, in which UV-B or UV-C can promote the accumulation of secondary metabolites in plants. For example, ultraviolet B radiation (UV-B; wavelength range 280–320 nm) is known to affect the synthesis of plant secondary metabolites and enhance the contents of phenols, terpenes, and anthocyanins in plant leaves [13, 14]. Tsurunaga et al. [15] showed that the quality of Tartary buckwheat could be improved and the accumulation of metabolites could be modified by altering the illumination received by plants. Exposure of Tartary buckwheat to UV-B irradiation, has been shown to induce the up-regulation of numerous genes in the phenylpropanol synthetic pathway, and enhances the accumulation of phenolic substances including flavonoids, tannins, and hydroxycinnamic acid derivatives produced via this pathway. The accumulation of flavonoids is associated with the reception of UV-B radiation in leaves and flowers, which have strong absorption peaks in the UV-B band, and flavonoid synthesis is assumed to play an important role in protecting plants from UV-B damage [16].

Phytohormones, such as jasmonic acid, salicylic acid, abscisic acid, and gibberellins (GA) are known to be important factor affecting the production of artemisinin in *A. annua* [17–21]. Among which GA has been reported up-regulation the expression of artemisinin biosynthesis genes, enhanced the accumulation of artemisinin, and promoted the formation of glandular trichomes. Gibberellins are diterpenoid plant hormones that mediate the regulation of multiple processes related to plant growth and development, including cell division and growth, seed germination, vascular bundle differentiation, and response to biotic or abiotic stresses. As diterpenoid acids, GA play an important role in promoting the biosynthesis of artemisinin [22]. Previous studies have demonstrated that artemisinin contents can be increased by three to four time in response to GA treatment, which also has the effect of increasing the number of glandular trichomes [23]. Moreover, it has been demonstrated that GA induces the expression of FDS, ADS, and CYP71AV1, which are key genes in the artemisinin biosynthetic pathway [18, 24], and exogenous GA has been reported to promote the conversion of artemisinic acid into artemisinin [25]. To date, however, there have been no studies that have investigated GA regulation of the molecular mechanisms underlying of artemisinin biosynthesis in *A. annua*.

It has been found that the activities of different hormones can be influenced to varying degrees by light, and thus light and hormones may jointly regulate various processes in plants [21, 26–28]. For example, Hao et al. [29] demonstrated the co-regulation of artemisinin biosynthetic pathway genes under light and jasmonic acid treatment, with the accumulation of artemisinin in response to jasmonic acid treatment being dependent on light, and identified eight transcription factors (TFs) as candidate genes regulating the cross-talk light and jasmonic acid signals in artemisinin biosynthesis. Along similar lines, we wished to determine whether ultraviolet B irradiation and phytohormone gibberellins could coordinately regulate the accumulation of artemisinin in *A. annua*.

Artemisinin is a sesquiterpene lactone compound with a peroxy bridge-group structure. In plants, terpenes are produced by means of two independent biosynthetic pathways, namely the mevalonate (MVA) pathway in the cytosol and the methylerythritol phosphate (MEP) pathway in plastids [30]. The upstream metabolic step in artemisinin biosynthesis occurs prior to the synthesis of farnesyl diphosphate (FPP), which serves as a substrate in the biosynthesis of different terpenoids via isopentenyl pyrophosphate (IPP). Amorpha-4,11-dienesynthase (ADS) catalyzed FPP to generate amorpha-4,11-diene [31], which is in turn oxidized to sequentially generate artemisinic aldehyde and artemisinic acid, under the catalysis of cytochrome P450 monooxygenase CYP71AV1 [32, 33]. The formation of dihydroartemisinic aldehyde is catalyzed by artemisinic aldehyde Δ11(13) reductase (DBR2), and this product is subsequently converted to dihydroartemisinic acid by aldehyde dehydrogenase 1 (ALDH1), which is eventually converted to artemisinin [2, 34].

In this study, we evaluated the combined effects of UV-B and GA on artemisinin biosynthesis, and based on transcriptome analysis, identified several candidate genes that may play regulatory roles in the synthesis of artemisinin. Our findings will provide important information for further studies on the regulation of artemisinin biosynthesis.

## Materials and methods

### Plant materials and culture conditions

The seeds of *A. annua* that were used in our study was collected in Hainan, which were a wild type. The wild seeds were collected and preserved in an accessible herbarium of Artemisinin Research Center, Institute of Chinese Materia Medica, China Academy of Chinese Medical Sciences. These seedlings were cultivated for 4 weeks under white light at 25 °C. The light intensity of the white light was 30 ± 5 μmol/m^2^ s, and the photoperiod was 16/8 h. The groups which were received neither GA nor UV-B treatment at 0, 6, 12, and 18 h, were used as control group. The UV-B light was UVB10.0-43B UVB tubes (275–320 nm Huaqiang Co. Ltd), 2 W/m^2^, measured using a UV-297 UV RADIOMETER (Dual channel) [35].The artemisinin contents were measured after 0 h, 6 h, 12 h and 18 h after light-emitting diode UV-B radiation and gibberellin (GA), respectively. The concentration of phytohormone gibberellin was 100 μmol/L. We collected the aboveground portion of the seedlings (which were cultivated for 4 weeks) treated with GA or UV-B. The samples were sampled immediately after 0 h, 6 h, 12 h and 18 h in different treatments, frozen in liquid nitrogen, and stored at − 80 °C for further analysis. Each treatment comprised three biological replicates (including 10 seedlings per repeat).

### Functional annotation

The total RNA from each sample for RNA-seq were extracted from seedling using RNAprep Pure Plant Kit (Tiangen, Beijing, China) and following the manufacturer’s instructions. Total RNA from each sample was used for Illumina sequencing at Novogene Bioinformatics Technology Co. Ltd. (Beijing, China). We prepared a total of 2 μg RNA to construct the sequencing libraries using NEBNext^®^ Ultra™ RNA Library Prep Kit for Illumina^®^ (NEB, MA, USA) with 28S/18S RNA ratio ≥ 1.8. The integrity of RNA was assessed using the Agilent 2100 Bioanalyzer (Agilent Technologies, CA, USA), and a minimum integrity number (RIN) value of 7 and 250–300 bp insertion element. The library was sequenced using an Illumina Xten sequencing system (Illumina Inc., San Diego, CA, USA).

### Transcriptome sequencing and data analysis

For exploration the possible molecular mechanism and regulatory mechanism of artemisinin biosynthesis induced by GA and UV-B, we collected the samples, including 12 transcriptome data from control group, GA, UV-B and GA + UV treatment groups. Illumina double-terminal sequencing was performed and high-quality clean reads were obtained. The raw data was submitted to NCBI ((PRJNA601869)). The clean reads were mapped to the *Artemisia annua* L. genome ASM311234v1 (https://www.ncbi.nlm.nih.gov/genome/?term=Artemisia+annua) [36] using STAR. HTSEQ 0.6.0 package was used to calculate gene expression level, which was expressed as fragments per kilobase of transcript per million fragments mapped (FPKM). Clusterprofile R package was used to compare DEGs with databases of Gene Ontology (GO), Kyoto Encyclopedia of Genes and Genomes (KEGG), so that these gene functions obtained were annotated and classified [37].

### Transcription factor identification and co-expression analysis

Transcription factor families (TFs) were distinguished using the PlantTFdb software. With the purpose of selecting the potential TFs regulating artemisinin biosynthesis, ADS (gene35372) and CYP71AV1 (gene56138) which were two greatly expressed genes, were applied to co-expression analysis, and a default value 0.5. From the above, two genes showing a Pearson correlation coefficient (r) more than 0.95 were considered as conspicuously co-expressed and were elected to establish a co-expression network by the Perl script and Cytoscape software version 3.7.1 [38].

### Quantitative Real-Time PCR (qRT-PCR) analysis of gene expression

The RNA samples were isolated by RNA Extraction Kit (Tiangen) and the first-strand cDNA was synthesized from 2 mg of RNA using PrimeScriptTM RT reagent Kit with gDNA Eraser (TaKaRA, Kusatsu, Japan). The selected pathway genes were identified by qRT-PCR using Rotor-GeneQ (Qiagen, Hilden, Germany) with TransStart Green qPCR SuperMix UDG (Transgene, Beijing, China). The gene-specific primers were designed by primer 6.0. Using two-step method for qPCR, and the genes expression quantity were analyzed by 2^−ΔΔCT^ method using the *A. annua* actin sequence as the internal reference gene (Additional file 1: Table S1). We all performed three biologic repetition for each sample. Each 20 μL reaction mixture contained 10 μL of TransStart Green qPCR SuperMix UDG, 1 μL of diluted cDNA, 0.4 μL of each primer (Forward/Reverse primer, 10 μM), 8.2 μL of double distilled water. The qPCR cycling conditions were as follows: 50 °C for 2 min; followed by 40 cycles of 94 °C for 5 s, and 60 °C 30 s in PCR strip tubes [39, 40].

### Analysis of co-expression modules based on WGCNA

Weighted Gene Co-Expression Network Analysis (WGCNA) package version 1.61 in the R software was used to build the gene co-expression networks from the normalized log2-transformed FPKM matrix [41]. The genes used for the network were based on the above RNA sequencing data from twelve samples of different treatment (control, GA, UV-B and GA + UV). To make the network show an approximate scale-free topology, an appropriate power value was determined with model fitting index R^2^ = 0.5 (Additional file 2: Figure S2) [42]. To classify genes with similar expression profiles into gene modules, average linkage hierarchical clustering was conducted according to the TOM-based dissimilarity measure with a minimum size (gene group) of 50 for the genes’ dendrogram [43].

### Liquid Chromatography–Mass Spectrometry (LC–MS) analysis of secondary metabolites

The powder of *A. annua* (0.1 g) was extracted with 1.0 mL pure methanol, and 0.4 mL of each extract was mixed and filtrated (0.22 μm) before LC–MS analysis. The artemisinin reference substance was precisely weighed (0.001 g) and applied to acquire the stock solution (1 mg/mL), which was melted by adding the corresponding volume of methanol. The analytical conditions were as follows: column was Agilent Elipse Plus C18 column (2.1 × 50 mm, 1.8 µm); solvent system was water (0.1% formic acid, A), acetonitrile (0.1% formic acid, B); time of gradient program was 0–3 min, 50–100%(B); 3–5 min, ~ 100%(B); flow rate was 0.2 mL/min; temperature was 35 °C; injection volume was 1 μL; Scanning mode was positive ion mode. The effluent was alternatively connected to an electrospray ionization (ESI)-triple quadrupole-linear ion trap (Q-TRAP)-MS or ESI-QqQ-MS/MS [39].

## Results

### Analysis of artemisinin content under different treatments

**Fig. 1:**
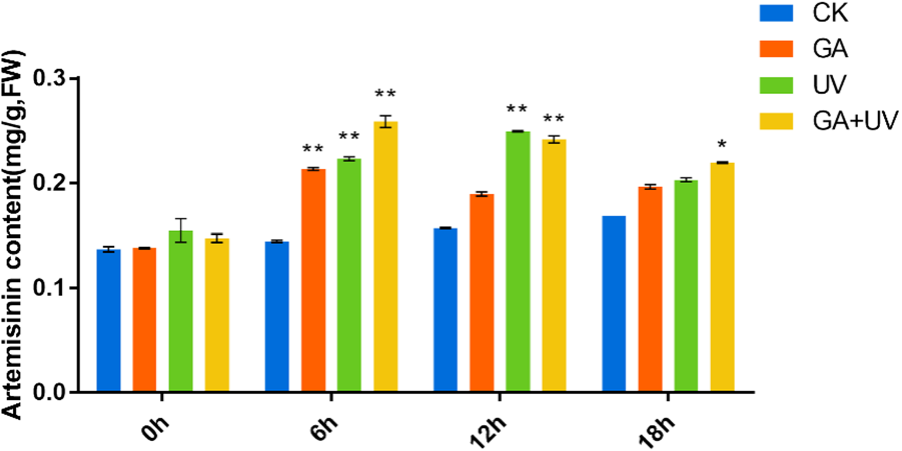
Artemisinin content in *Artemisia annua* L. plants treated with gibberellin (GA), ultra-violet-B irradiation (UV), or a combination of gibberellin and ultra-violet-B irradiation

To assess the effects of GA or UV-B on artemisinin biosynthesis, we treated *A. annua* seedlings with either GA or UV-B radiation separately or subjected plants to a combined GA + UV treatment and determined the artemisinin concentrations in these plants after 0, 6, 12, and 18 h of the different treatments. In addition, the groups, which were received neither GA nor UV-B treatment at 0, 6, 12, and 18 h, were used as control group. Compared with the control plants, we detected an increase in artemisinin concentrations in those *A. annua* seedlings exposed to UV-B. Moreover, artemisinin concentrations in plants subjected to the GA + UV treatment were found to be notably higher than those in plants exposed to UV-B only (Fig. 1). As is shown in Fig. 1, artemisinin concentrations increased in a time-dependent manner in response to UV-B irradiation; however, a gradual decrease in the content of artemisinin was noted at 18 h, which we presume to be associated with UV-B-induced damage. In addition, we found that artemisinin concentrations under GA + UV treatment were significantly higher than that control group at 6 h and 12 h. Moreover, at 6 h, artemisinin concentrations in plants subjected to the GA + UV treatment were found to be significantly higher than those in plants treated with either UV-B or GA treatment, whereas no significant difference was observed between GA + UV and GA treatments at 18 h. Collectively, these results indicated that GA + UV and UV-B can enhance the artemisinin content of *A. annua* at 6 h after the initiation of treatment.

### Identification of differentially expressed genes and functional annotation and classification

**Fig. 2:**
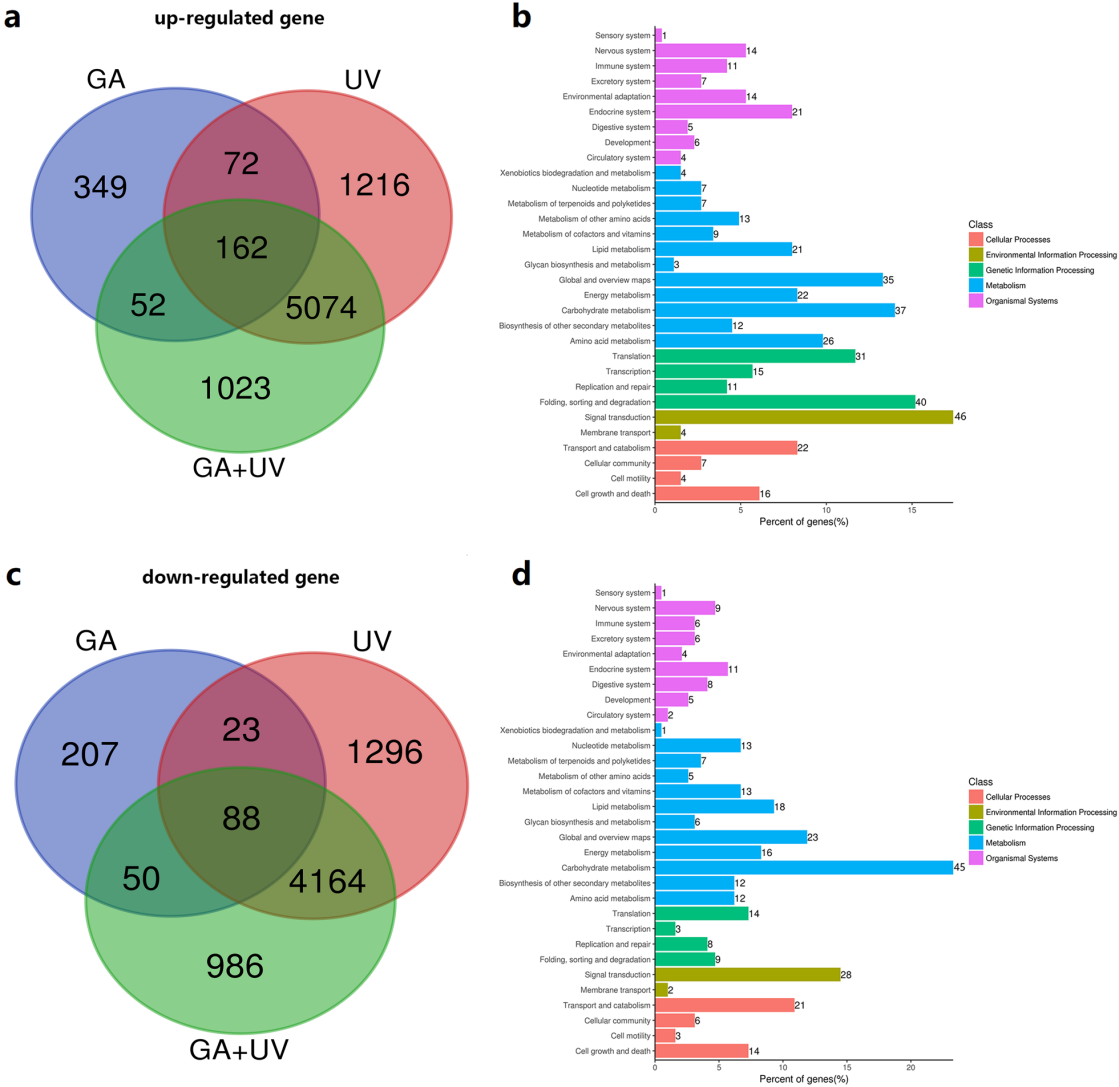
Numbers of differentially expressed genes (DEGs) and KEGG pathway enrichment analysis of the DEGs in the different treatments of gibberellin (GA), ultra-violet-B irradiation (UV), and a combination of GA and UV. **a** Venn Diagram showing up-regulation DEGs genes; **b** KEGG analysis of up-regulated genes; **c** Venn Diagram showing down-regulation DEGs genes; **d** KEGG analysis of down-regulated genes

Given that GA and UV-B treatments had significant effects on the accumulation of artemisinin at 6 h, we performed transcriptome analysis using samples collected at 6 h after treatment initiation. We accordingly identified a total of 14,762 differentially expressed genes (DEGs) in *A. annua*, among which 635, 6524, and 6311 genes were up-regulated in plants treated with GA, UV-B, and GA + UV, respectively (Fig. 2a). Comparatively, 368, 5571, and 5288 genes were down-regulated in GA, UV-B, and GA + UV treatments, respectively (Fig. 2c). Comparison between the GA + UV and GA treatments revealed 214 and 138 genes that were up- and down-regulated, respectively. Whereas 5236 and 4252 genes were up- and down-regulated between the GA + UV and UV-B treatments, 234 and 111 genes were up- and down-regulated between the GA and UV-B treatments. Furthermore, we found that 162 and 88 genes were simultaneously up- and down-regulated, respectively in response to both GA, UV-B, and GA + UV treatments (Fig. 2, Additional file 3: Table S2).

To identify differences in the processes affected by treatment with UV-B and GA, we subjected the DEGs to GO and KEGG pathway analyses. The proportions of genes enriched in the three main GO categories for GA + UV, UV-B, and GA treatments are summarized in Additional file 4: Figure S1. A total of 5584 up-regulated DEGs were annotated into GO database and were classified into 60 functional groups, including biological process, molecular function and cellular component (Additional file 4: Figure S1). Within the biological process, ‘biosynthetic process’ (GO: 0009058; 620 genes) and ‘cellular nitrogen compound metabolic process’ (GO: 0034641; 461 genes) and ‘response to stress’ (GO: 0006950; 384 genes) were predominant. The terms ‘cellular component’ (GO: 0005575; 1916 genes) was the most common in the cellular component category. In the molecular function category, the two main groups were the ‘ion binding’ (GO: 0046914; 2220 genes) and ‘molecular function’ (GO: 0003674; 1171 genes). To identify 4501 down-regulated DEGs were annotated in the GO database (Additional file 4: Figure S1B). Within the biological process, ‘biosynthetic process’ (GO: 0009058; 562 genes) and ‘cellular nitrogen compound metabolic process’ (GO: 0034641; 344 genes) were predominant. The terms ‘cellular component’ (GO: 0005575; 1891 genes) was the most common in the cellular component category. In the molecular function category, the two main groups were the ‘ion binding’ (GO: 0046914; 1466 genes) and ‘molecular function’ (GO: 0003674; 1049 genes).

The analysis of KEGG pathway was performed to distinguish metabolic pathways (Fig. 2b, d). The analysis of up-regulated genes, within the organismal systems process, ‘Endocrine system’ (21), metabolism process, ‘Global and overview maps’ (35) and ‘Carbohydrate metabolism’ (37), genetic information processing, ‘translation’(31) and ‘folding, sorting and degradation’ (40), environmental information processing, ‘signal transduction’(46), cellular processing, ‘transport and catabolism’ (22). The KEGG pathway analysis of down-regulated genes, within the organismal systems process, ‘Endocrine system’ (11), metabolism process, ‘Global and overview maps’ (23) and ‘Carbohydrate metabolism’(45), genetic information processing, ‘translation’(14) and ‘folding, sorting and degradation’(9), environmental information processing, ‘signal transduction’(28), cellular processing, ‘transport and catabolism’ (21).

### Expression analysis of the genes involved in artemisinin biosynthesis

**Fig. 3:**
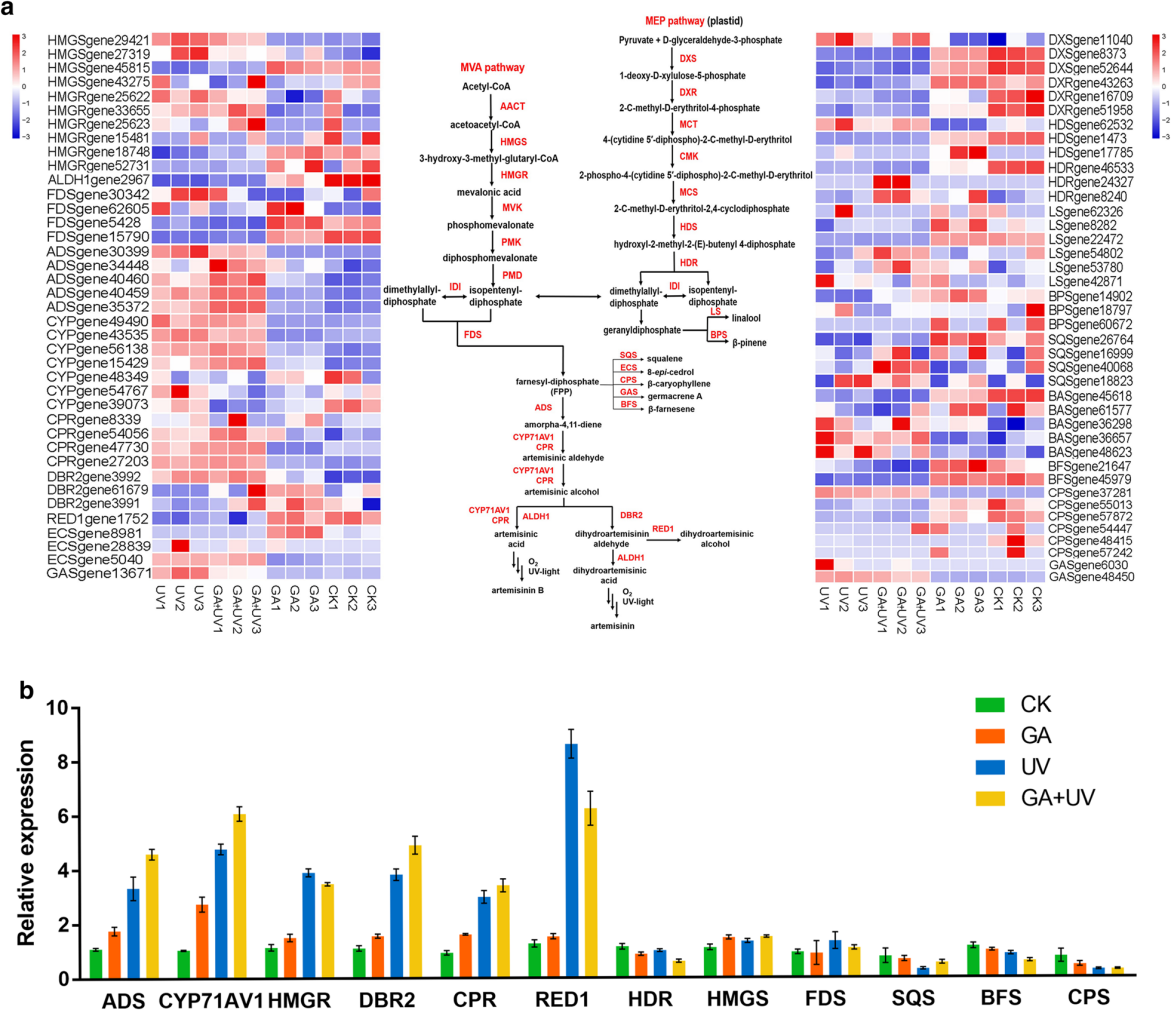
**a** Heatmaps showing variations in the expression of genes involved in artemisinin biosynthesis. **b** Validation of RNA sequencing results by quantitative real-time quantitative PCR (qRT-PCR) of selected genes

Artemisinin is synthesized via multiple enzymatic steps in the sesquiterpene pathway. Our transcriptome analysis revealed that numerous differentially expressed genes were annotated as artemisinin biosynthetic genes. Among these, we analyzed the transcript levels of 21 artemisinin biosynthesis-related structural genes (Fig. 3a). In the mevalonate (MVA) pathway, 3-hydroxy-3-methyl-glutaryl CoA synthase (HMGS) and 3-hydroxy-3-methyl-glutaryl CoA reductase (HMGR) catalyze the two-step conversion of acetoacetyl-COA to mevalonic acid, and in the present study, we found the expression of HMGS was almost the same under GA, UV-B and GA + UV treatments, indicating that GA and UV-B does not promote the expression of HMGS. In contrast, the expression of HMGR was 3.42-fold higher in response to UV-B treatment compared with the control, and the response to GA + UV treatment was almost identical to that in induced by UV-B (Fig. 3b).

Farnesyl diphosphate (FPP), a precursor of amorpha-4, 11- diene, and other sesquiterpenes in *A. annua*, can be metabolized by different sesquiterpene synthases in competing pathways. Squalene synthase (SQS) is a key enzyme in the biosynthesis of sterols and triterpenes, which are competing pathways with respect to artemisinin biosynthesis. Zhang et al. [44] showed that inhibition of the expression of SQS by hairpin RNA-mediated RNAi increased artemisinin content by approximately 3.14-fold in transgenic plant lines. β-caryophyllene synthase (CPS) catalyzes the conversion of FPP to β-caryophyllene, which is also a competing pathway with respect to artemisinin biosynthesis, and it has previously been shown that down-regulation of CPS in *A. annua* resulted in a 54.9% increase in the content of artemisinin in transgenic plants [45]. In a further biosynthetic pathway that competes with that of artemisinin, β-farnesene synthase (BFS) catalyzes the conversion of FPP to β-farnesene [46]. In the present study, we compared with the control treatment, the expression of SQS, CPS, and BFS was down-regulated in response to UV-B and GA + UV treatments (Fig. 3b). Accordingly, we speculate that the observed increases in artemisinin content under these treatments could be explained by the down-regulation of enzymes in competing sesquiterpene pathways.

Amorpha-4,11-dienesynthase (ADS) is the first rate-limiting enzyme in the artemisinin biosynthetic pathway, and it has previously been observed that artemisinin contents were increased by approximately 59% in ADS-overexpressing transgenic plants [47]. Furthermore, overexpression of the CYP71AV1 and CPR genes in transgenic plants resulted in artemisinin contents that were almost 38% higher than those in non-transgenic *A. annua* [48]. Compared with the control treatment at 6 h, we found in the present study the GA, UV-B, and GA + UV treatments promoted 1.61-fold, 3.05-fold and 4.19-fold increases in the expression of ADS, respectively. Similarly, treatment with GA, UV-B, and GA + UV also led to 2.63-fold, 4.57-fold and 5.82-fold increases in the expression of CYP71AV1 (Fig. 3b). Interestingly, we found that the expression level of ADS, CYP71AV1, DBR2 and CPR were up-regulated to a greater extent under GA + UV treatment than in response to UV-B treatment alone (Fig. 3). In order to validate the recorded levels of DEGs, we performed quantitative real-time PCR analysis of 9 selected genes. The resulting qRT-PCR expression profile were found to show a positive correlation with the transcriptome data (Fig. 3b).

### Identification of co-expressed transcription factors and artemisinin biosynthesis genes

**Fig. 4:**
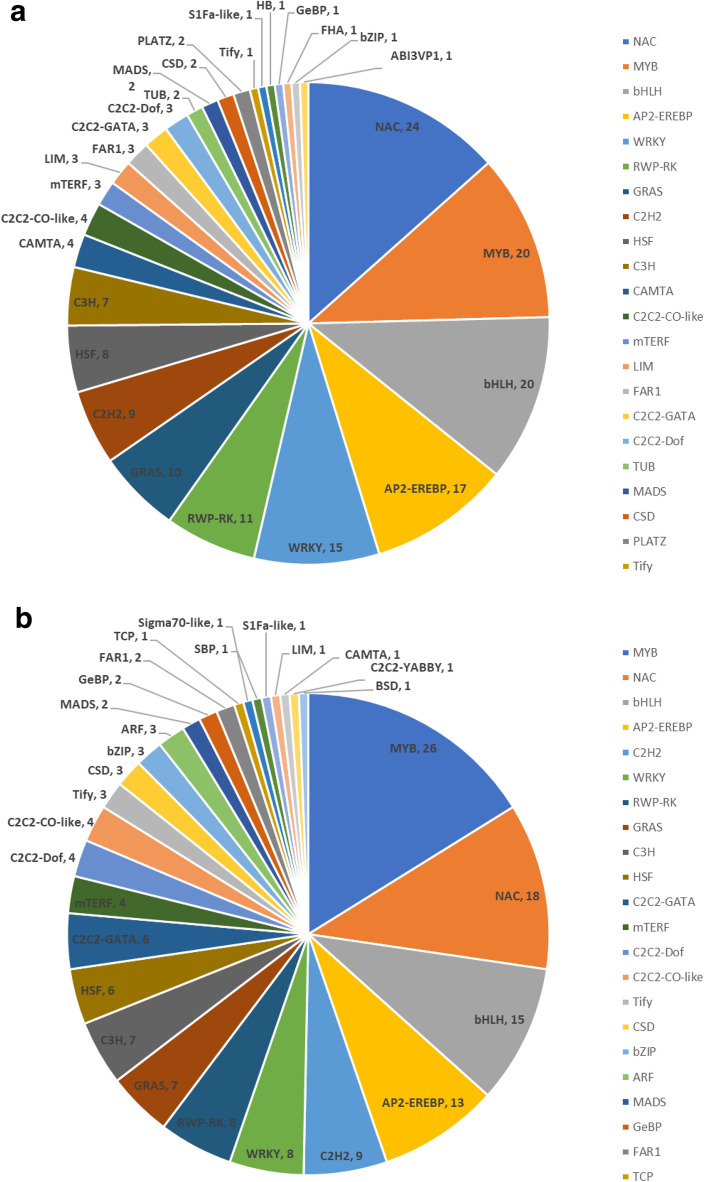

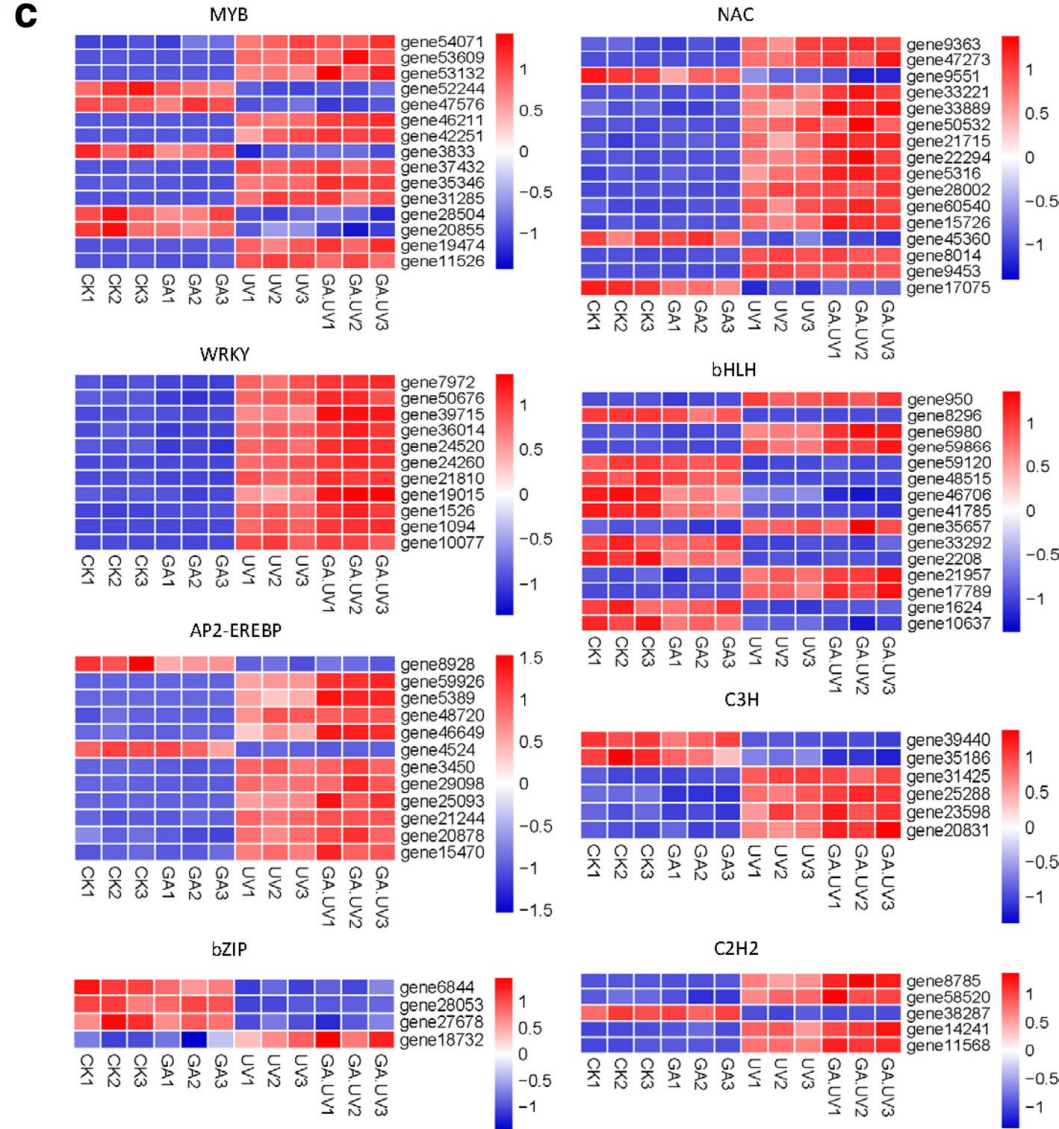
Distribution of transcription factor (TF) families in the co-expression results. ADS (**a)** and CYP71AV1 (**b**) (|r| > 0.95). The size of node circle is positively correlated with the number of the interacting gene partners. **c** Heat map diagrams of factor (TF) families in the co-expression results. MYB, MYB proteins; NAC, NAM/ATAF/CUC; WRKY, WRKY proteins; bHLH, basic helix-loophelix; bZIP, basic region/leucine zipper; AP2-EREBP, AP2/ERF domain-containing protein; C2H2, C2H2 zinc-finger proteins; C3H, Cys3His zinc finger

Among the DEGs detected, we identified a total of 1295 transcription factors (TFs), which were subjected to co-expression analysis to identify those TFs involved in the regulation of genes associated with artemisinin biosynthesis. We accordingly found that 179 and 161 TFs were co-expressed with gene35372 (ADS) and gene56138 (CYP71AV1) (|r| > 0.95), respectively. Among the TFs co-expressed with CYP71AV1, 26, 18, 15, and 13 were MYB, NAC, bHLH, and AP2-EREBP TFs, respectively (Fig. 4). Similarly, 24, 20, 20, 17, and 15 NAC, bHLH, MYB, AP2-EREBP, and WRKY TFs, respectively, were co-expressed with ADS (Additional file 5: Table S3). Consistently, these TFs have previously been reported to play significant roles in regulating the expression of artemisinin biosynthesis genes. In this study, according to high degree value and high expression level, a total of 84 co-expressed transcription factors were identified, which were considered as the presumed regulators of artemisinin biosynthesis responding to GA + UV and co-expressed with ADS and CYP71AV1 genes (Fig. 4c). Transcription factors included MYB (MYB proteins), NAC (NAM/ATAF/CUC), WRKY (WRKY proteins), bHLH (basic helix-loophelix), bZIP (basic region/leucine zipper), AP2-EREBP (AP2/ERF domain-containing protein), C2H2 (C2H2 zinc-finger proteins), C3H (Cys3His zinc finger).

### WGCNA and Identification the functional annotation of artemisinin‑related modules

**Fig. 5:**
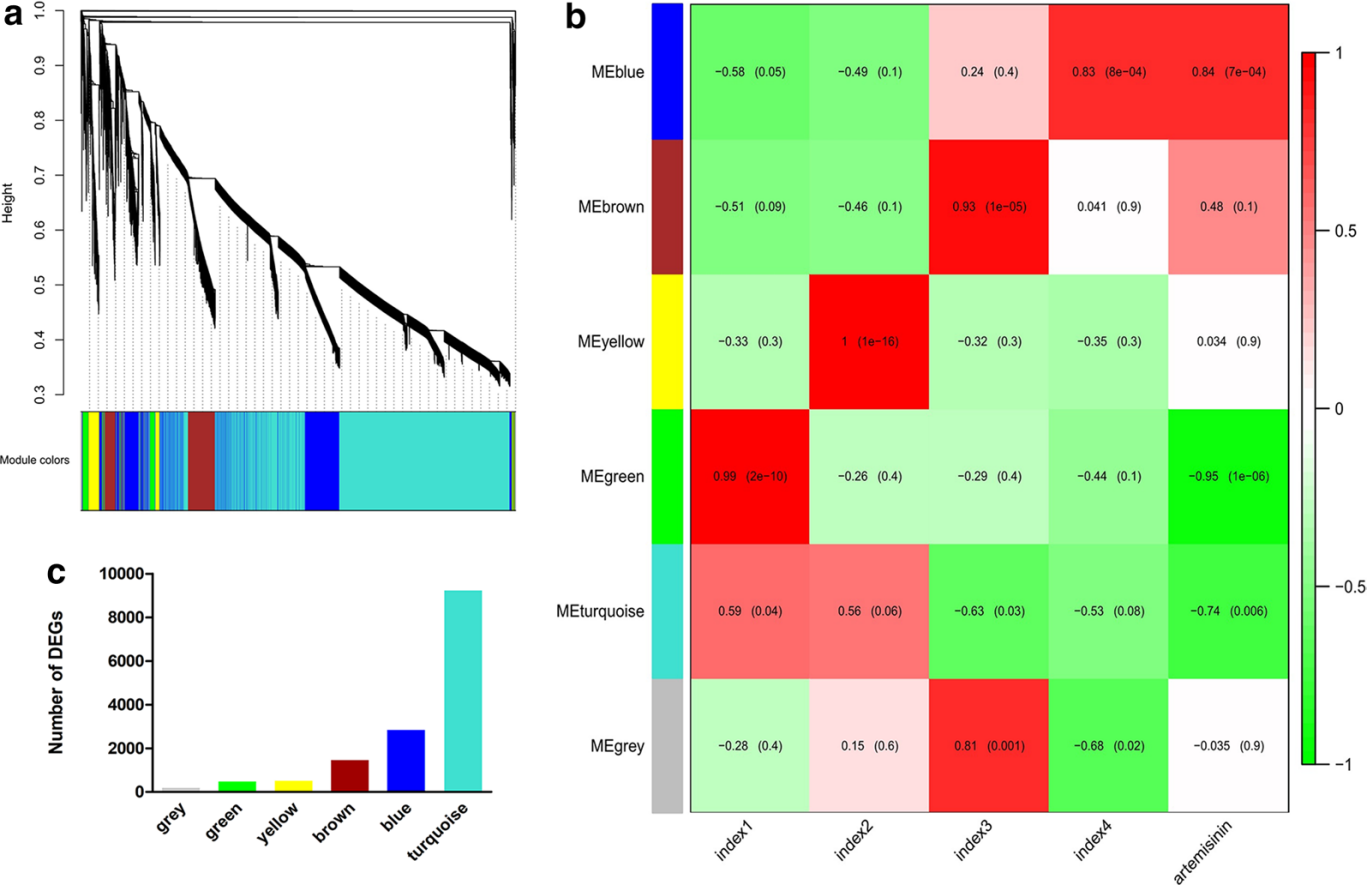
Detection of co-expressed modules among the artemisinin‑related genes based on weighted gene co-expression network analysis. **a** Dendrogram showing the different genes clustered into co-expressed modules. **b** Module-trait associations. Association between artemisinin-related co-expressed modules with UV-B light and phytohormone GA treatments in *A. annua*. **c** Number of assigned DEGs to the different modules

**Fig. 6:**
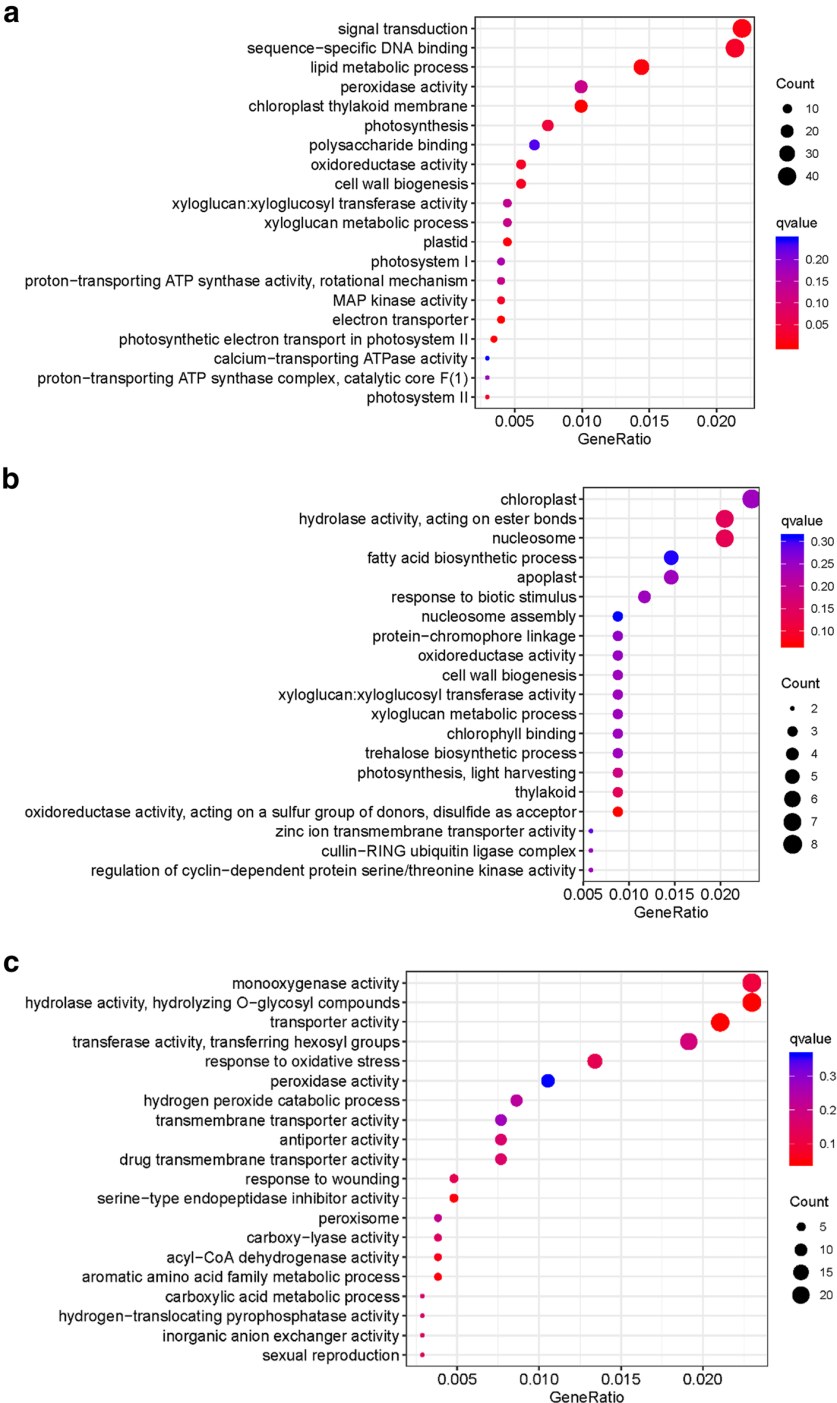
GO enrichment analysis of the co-expressed gene modules. **a** GO enrichment analysis of blue module. **b** GO enrichment analysis of brown module. **c** GO enrichment analysis of yellow module

In order to determine the different modules of co-expressed genes, we performed weighted gene co-expression network analysis (WGCNA) on the DEGs. WGCNA divided the 14582 DEGs into six different modules, namely, MEblue, MEbrown, MEyellow, MEgreen, MEturquoise and MEgray, which contained 2829, 1450, 511, 482, 9233, and 34 genes, respectively (Fig. 5a, c; Additional file 6: Table S4). Evaluation of the associations between the detected modules and artemisinin content indices revealed differences in the relationships between the six modules and artemisinin content. With respect to GA + UV treatment, we found that MEblue module was significantly correlated with artemisinin content (r = 0.83), suggesting that the DEGs in these modules should be up-regulated to promote an increase in artemisinin in response to GA + UV treatment. To examine the biological processes associated with the blue and green modules explored by WGCNA, we performed GO enrichment analysis (Fig. 6). We accordingly found that blue module-related genes stand for the basal defense of *A. annua* as evidenced by the enrichment of GO terms such as “signal transduction” and “sequence-specific DNA binding” (Fig. 6a). In contrast, with respect to control (CK) and GA treatment in the MEgreen module, were negatively correlated with artemisinin content (r = 0.99, − 0.26, respectively), suggesting that the DEGs in these modules should be down-regulated to promote an increase in artemisinin content under control or GA treatment, enriched in the category “chloroplast” (Additional file 8: Figure S4A). Further, we observed that the DEGs are associated with distinct responses according to the artemisinin content, highlighting the key roles played by regulatory genes in determining the artemisinin‑related processes (Fig. 5b). These results further support the premise that the co-expressed gene modules of the artemisia DEGs play different roles with respect to artemisinin‑related processes.

To understand the gene interactions within each module, we established the network of the detected co-expressed modules using Cytoscape 3.7.1 software, in order to distinguish candidate hub genes. In the above studies, the blue, brown, and yellow modules had higher responses to UV light or gibberellins, the plant hormone gibberellin and UV + GA treatments, respectively. So, the hub gene which was related to artemisinin, was found in the blue, brown, and yellow modules. Genes had clusters in the blue, brown, and yellow modules, each with a different number of genes (Additional file 7: Figure S3). TFs were represented by different node colors, except that sky blue represents functional genes and the size of node circles was positively correlated with the number of interacting genes. Hub gene is the largest gene of nodes in network. In the blue module, we found several hub genes, including gene17498 (cytochrome P450 mono-oxygenase), gene20621 (plastid delta12-fatty acid acetylenase), gene63203 (photosynthetic reaction centre L/M Photosystem antenna protein-like protein plastid) and gene46617 (photosystem I P700 apoprotein A chloroplast) etc. (Additional file 7: Figure S3A). In the brown module, the hub genes detected are gene3771 (cytochrome P450), gene30537 (photosystem II 5kD protein), gene10647 (Dehydrogenase E1 component) and gene50594 (hypothetical protein) (Additional file 7: Figure S3B). The yellow module contains complex clusters of genes, we observed two gene clusters linked by the gene45686 (leucine-rich repeat domain L domain-like protein). Namely, there are multiple biological functions in this module (Additional file 7: Figure S3C). In addition, key TFs are also present in the blue, brown and yellow module, which may play the important regulatory role, including gene39715 (WRKY51) in the blue module, gene31207 (WRKY) in the brown module, gene25838 (AP2-EREBP) and gene760 (AP2-EREBP) in the yellow module. However, there is no gene encoding TFs in the green, turquoise and grey module (Additional file 7: Figure S3, Additional file 8: Figure S4). We infer that these hub genes in their modules may be potential genes that regulate and enhance the specific response to UV light and gibberellin treatments in *A. annua*.

## Discussion

Gibberellins are important diterpenoid acid plant hormones that play roles in promoting the biosynthesis of artemisinin [29, 49]. In addition, to phytohormones, it has also been found that light has an influence on the accumulation of artemisinin [39]. Ultraviolet B radiation is known to affect the synthesis of secondary metabolites in plant leaves [23, 50, 51]. Moreover, the effect of other plant hormones such as methyl jasmonate on artemisinin has been shown to be light dependent. However, to date, there have been no studies that have sought to identify the DEGs related to GA-promoted artemisinin biosynthesis in plants subjected to UV-B irradiation, and little is known regarding the related signaling pathways. To the best of our knowledge, the present study is the first study to investigate the transcriptional changes associated with artemisinin accumulation in response to combined treatment with GA and UV-B.

In this study, we revealed that UV-B light and GA facilitated the accumulation of artemisinin in *A. annua* seedling, as determined by LC–MS analyses. The results highlighted the important role played by UV-B light in terms of enhancing the content of artemisinin. Under GA treatment, the artemisinin content was lower than produced in response to UV-B light, although higher than that in the control group. Interestingly, in plants treated with a combination of GA and UV-B, the content of artemisinin was clearly higher than that obtained in plants exposed to UV-B light only. Thus, UV-B was shown to have a clear effect on the biosynthetic of artemisinin, promoting an increase in the content of this plant secondary metabolite. Moreover, it was also observed to augment GA-promoted artemisinin biosynthesis. Collectively, these results thus indicate that combined treatment with GA and UV-B treatment can enhance the content of artemisinin in *A. annua* plants.

**Fig. 7:**
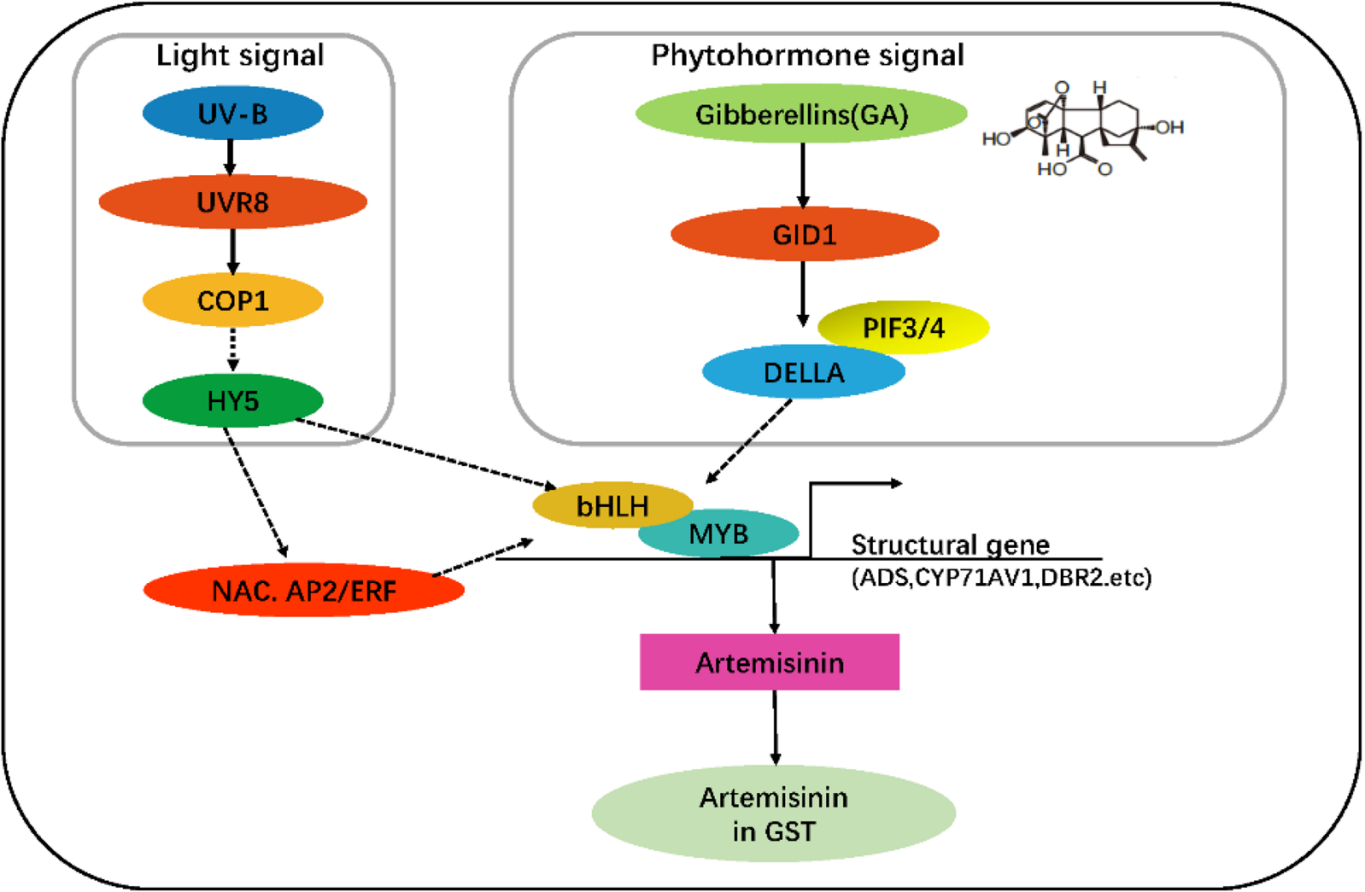
A proposed model of UV-B and GA signal nodes coordinately regulated key genes in the artemisinin metabolism pathway

Based on a comparison of the different treatments assessed in the present study, we identified DEGs associated artemisinin biosynthesis. Further, GO annotations revealed that the genes up-regulated in response to UV-B and GA treatments are closely related to biosynthetic processes and responses to stress. Moreover, results of KEGG analysis showed that, in addition to artemisinin synthesis, exposure to UV-B light had notable effects on the growth and development of *A. annua*. Venn diagrams depicting the numbers of genes that were up-regulated and down-regulated in response to UV-B light and GA treatments show the commonly and uniquely expressed genes under each treatment. UV-B is known to interact with COP1, which in turn promotes the expression of the HY5 gene, a key factor linking light- and hormone-mediated effects, although the underlying mechanism has yet to be elucidated [52]. A close relationship between GA and light has, however, been shown to be mediated by the interaction between DELLA protein and PIF3/PIF4 [29, 49]. DELLA was degraded in a high GA level. It is beneficial for PIF3/4 to bind to the promoter of downstream genes. The seedling grown under light has a lower GA level and DELLA accumulation, which interacted with PIF3/4 and inhibition the binding of the promoter of downstream genes, which further hindered the regulation of artemisinin. HY5 and DELLA are often associated with plant growth and development, which are important signal nodes for UV-B and GA, respectively [23, 53, 54]. We hypothesized that these UV-B and GA signal nodes affect key genes in the artemisinin metabolism pathway, and future studies are expected to focus on the on the mechanisms underlying the regulation of artemisinin biosynthesis via the HY5 and DELLA signal nodes (Fig. 7).

Previous studies have shown that phytohormones, such as methyl jasmonate, abscisic acid, salicylic acid, and GA, have a positive influence with respect to artemisinin production in *A. annua*. Furthermore, some studies have reported that UV-B light can increase the concentration of artemisinin and up-regulate the expression of the artemisinin biosynthetic genes AaADS and AaCYP71AV1, and related WRKY TFs [22]. Given that we found that the expression of artemisinin biosynthetic genes can be affected by GA and UV-B treatments, we sought to determine whether there is cross-talk between the effects mediated by these two treatments. Heatmap and qRT-PCR analyses revealed that treatment of *A. annua* seedlings with a combination of GA and UV-B can enhance the accumulation artemisinin by coordinately promoting the expression of genes involved in artemisinin biosynthesis, including ADS, CYP71AV1, and DBR2, and inhibiting genes such as SQS and BFS that function in competing biosynthetic pathways. This synergistic interaction elevated the expressions of some key genes in artemisinin biosynthetic to levels that were higher than those induced in response to either UV-B or GA treatment alone, e.g., ADS, CYP71AV1, DBR2, CPR genes. We accordingly concluded that these two treatments might enhance artemisinin levels via the coordinated regulation mechanism, and further investigated this possibility using transcriptome sequencing.

TFs play important roles in regulating the synthesis and accumulation of secondary metabolites in the plants. Transcriptional regulation is, nevertheless, a complex process, as a single TF can potentially regulate the expression of multiple genes in a single or multiple metabolic pathway [55]. TFs indirectly regulate artemisinin biosynthesis and control the accumulation of specific secondary metabolites by activating or inhibiting the expression of target genes, and it has been reported that TFs such as AaWRKY1 [47, 56], AaERF1 [57], AaERF2 [58], AaORA [59], AabZIP1 [60], AaMYC2 [61], AaMYB106 [62], AaNAC1 [63], and AabHLH1 [64] can affect the content of artemisinin by regulating the expression of the key genes ADS and CYP71AV1 in the artemisinin biosynthesis pathway. In recent years, transcriptome sequencing has been widely used to analyze and identify various TF families in medicinal plants, including Panax ginseng, Salvia miltiorrhiza, and Tartary buckwheat [65–67]. Previously, it has been demonstrated that the AaAOC gene regulates artemisinin content by modifying jasmonic acid production, whereas AaPYL9 is known to regulate artemisinin content by affecting abscisic acid signaling [68]. Furthermore, Hao et al. [69] have demonstrated that light-induced artemisinin biosynthesis in *A. annua* is regulated by the bZIP transcription factor AaHY5. To date, however, only a relatively few TFs associated with phytohormone- and light-mediated induction of artemisinin accumulation have been identified and characterized. Accordingly, further transcriptome sequencing is warranted to identify other TFs involved in the phytohormone and light signaling associated with artemisinin biosynthesis.

Most of the relevant TFs identified to date, including WRKY, bHLH, NAC, AP2/ERF, and bZIP, appear to regulate artemisinin biosynthesis via modifications of hormone signals. In contrast, the identity of those TFs induced by both UV-B light and hormones remains unclear. Our co-expression analysis indicated the likelihood that TF activation is induced by both UV-B light and GA, which in turn might promote the regulation of artemisinin biosynthetic gene expression. Our results revealed that MYB TFs were the most frequent TFs associated with artemisinin biosynthesis, and that their expression was induced by both UV-B light and GA (Fig. 4). In addition, we identified 30 NAC TFs and 25 AP2/ERF TFs, which are typically associated with secondary metabolism and resistance to abiotic stresses in plants. We also detected 26 bHLH TFs and 4 bZIP TFs, among the latter of which is HY5, which functions as a central point in the light signal transduction pathway. Furthermore, phytochrome-interacting factors belonging to the bHLH family are also significant light signal-related TFs that are implicated in the regulation of multiple development processes. In the present study, we also identified 17 WRKY TFs, which are typically associated with the response to GA + UV light stress. We believe that the preliminary identification of these candidate TFs associated with the transcriptional regulation of artemisinin by both UV-B light and GA will provide a valuable foundation for further in-depth studies on the mechanisms underlying the regulation of artemisinin synthesis.

## Conclusions

In this research reported that both UV-B light and GA treatment observably induced the expression of genes in artemisinin biosynthesis, resulting in the increase of artemisinin concentration. The expression of ADS and CYP71AV1 genes, and relevant NAC transcription factors were significantly up-regulated during the biosynthesis of artemisinin. In addition, the study revealed that UV-B light and phytohormone gibberellins coordinately promoted the activation of many artemisinin biosynthesis synthetases genes and inhibited the expression of synthetases in competitive pathway, as a result the accumulation of artemisinin content could be positively increased in *A. annua*. In this study, according to the high degree value and high expression level, a total of 84 co-expressed transcription factors were identified, which were considered as the presumed regulators of artemisinin biosynthesis responding to GA + UV and co-expressed with ADS and CYP71AV1 genes. The co-expression was analysis revealed that the selected MYB and NAC TFs might have regulated the artemisinin biosynthesis gene expression by UV-B light and phytohormone gibberellins. Weighted gene co-expression network analysis revealed that GA + UV in blue modules was positively correlated with artemisinin synthesis. We established the network to distinguish candidate hub genes in blue modules should be up-regulated to enhance artemisinin synthesis in response to GA + UV treatment.

## Supplementary information

**Additional file 1: Table S1.** List of the primers used for qRT-PCR experiments in Artemisia annul.

**Additional file 2: Figure S2.** Detection of co-expressed modules based on weighted gene co-expression network analysis. (A) Clustering of the modules. (B) Analysis of network topology through different soft-threshold limits.

**Additional file 3: Table S2.** Expression value (FPKM) of the 250 differentially expressed genes (DEGs) in response to both GA, UV, and GA+UV treatments in artemisia and their predicted function (162 up-regulation DEGs genes, 88 down-regulation DEGs genes). CK = control group, GA = gibberellin, UV = ultra-violet-B irradiation, GA+UV = combination of gibberellin and ultra-violet-B irradiation.

**Additional file 4: Figure S1.** Functional annotation of all of the assembled unigenes into biological process, cellular component, and molecular function categories within the gene ontology (GO) database.(A) Gene ontology (GO) database of up-regulated genes; (B) Gene ontology (GO) database of down-regulated genes. KEGG pathway enrichment analysis of the annotated DEGs in the different treatments. (C) Number of differentially expressed genes detected between GA, UV and GA+UV treatments.

**Additional file 5: Table S3.** Distribution of transcription factor (TF) families in the co-expression results. 179 TFs and 161 TFs were co-expressed with ADS and CYP71AV1 (|r| > 0.95), respectively.

**Additional file 6: Table S4.** Repartition of the artemisinin-related genes after different treatments into the six co-expressed modules (Blue, Green, Brown, Yellow, Turquoise and Grey) detected by WGCNA.

**Additional file 7: Figure S3.** Weighted gene co-expression network analysis the co-expressed network of blue module (A), brown module (B) and yellow module (C), green module (D), turquoise module (E) and grey module (F).

**Additional file 8: Figure S4.** GO enrichment analysis of the co-expressed gene modules. (A) GO enrichment analysis of green module. (B) GO enrichment analysis of turquoise module. (C) GO enrichment analysis of grey module.

## Data Availability

The datasets used in this study are available from the corresponding author upon reasonable request.

## Abbreviations

MVA pathway: The mevalonate pathway
MEP pathway: The 2-C-methyl-d-erythritol 4-phosphate pathway
AACT: Acetoacetyl-CoA thiolase
HMGS: 3-hydroxy-3-methyl-glutaryl coenzyme A synthase
HMGR: 3-hydroxy-3-methyl-glutaryl coenzyme A reductase
MVK: Mevalonate kinase
PMK: Phosphomevalonate kinase
PMD: Diphosphomevalonate decarboxylase
IDI: Isopentenyl diphosphate isomerase
FDS: Farnesyl diphosphate synthase
DXS: 1-Deoxy-d-xylulose-5-phosphate synthase
DXR: 1-Deoxy-d-xylulose-5-phosphate reductoisomerase
MCT: 2-C-methyl-d-erythritol-4-(cytidyl-5-diphosphate) transferase
CMK: 4-cytidine 5′-diphospho-2-C-methyl-d-erythritol kinase
MCS: 2-C-methyl-d-erythritol-2,4-cyclodiphosphate synthase
HDS: Hydroxy-2-methyl-2-(E)-butenyl 4-diphosphate synthase
HDR: Hydroxy-2-methyl-2-(E)-butenyl 4-diphosphate reductase
LS: Linalool synthase
BPS: β-Pinene synthase
SQS: Squalene synthase
BAS: β-Amyrin synthase
BFS: β-Farnesene synthase
CPS: β-Caryophyllene synthase
ECS: Epi-cedrol synthase
GAS: Germacrene A synthase
ADS: Amorpha-4,11-diene synthase
ALDH1: Aldehyde dehydrogenase 1
CPR: Cytochrome P450 reductase
CYP71AV1: Cytochrome P450 monooxygenase
DBR2: Artemisinic aldehyde Δ11(13) reductase
RED1: Dihydroartemisinic aldehyde reductase
DEGs: Differentially expressed genes
GST: Glandular secretory trichomes
