# Supplementary material for: Transcriptome analyses revealed the ultraviolet B irradiation and phytohormone gibberellins coordinately promoted the accumulation of artemisinin in Artemisia annua L
Source: Chin Med. 2020 Jul 1;15:67. doi: 10.1186/s13020-020-00344-8 (PMC7329506; doi:10.1186/s13020-020-00344-8)

**Figure S2.** Detection of co-expressed modules based on weighted gene co-expression network analysis. **(A)** Clustering of the modules**. (B)** Analysis of network topology through different soft-threshold limits.


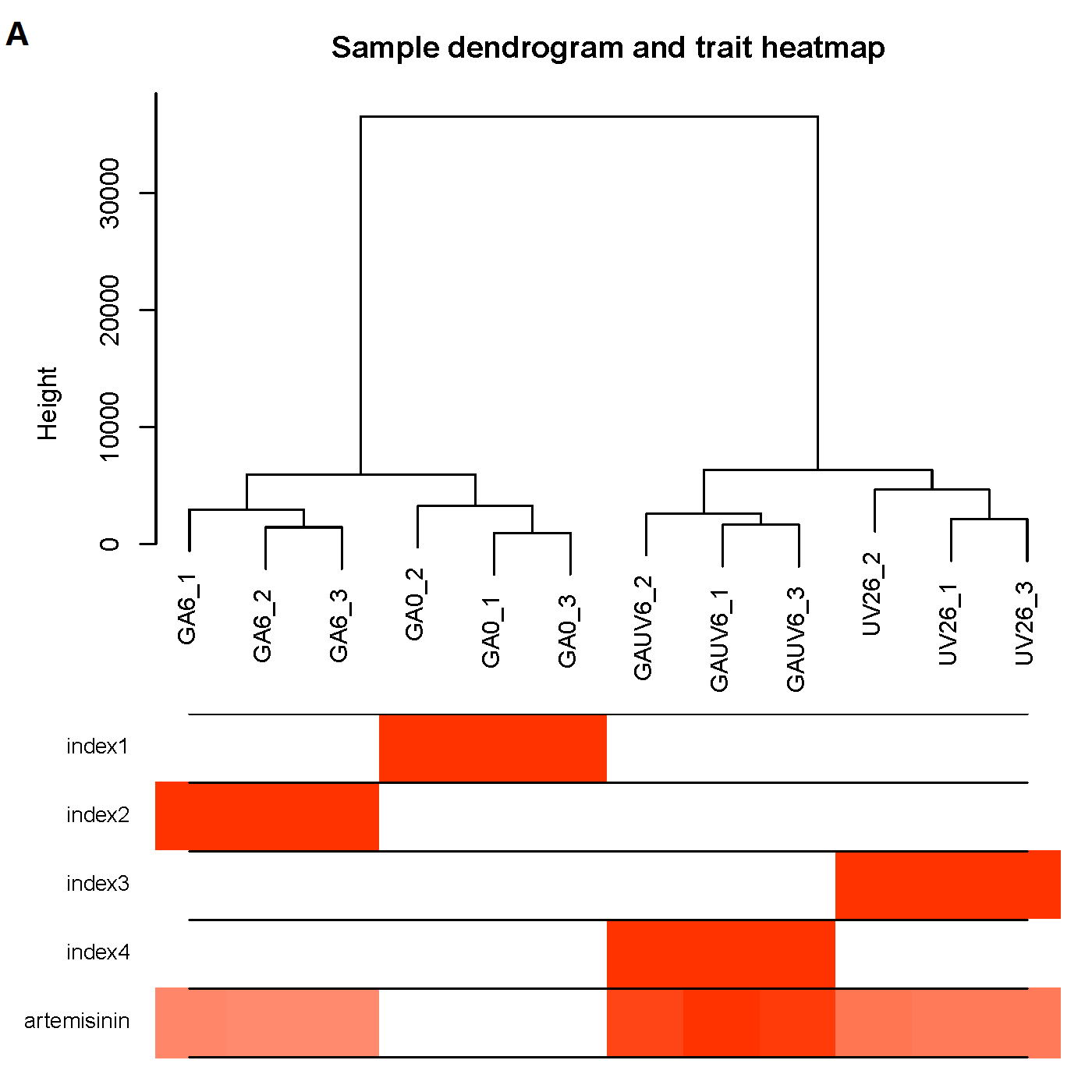


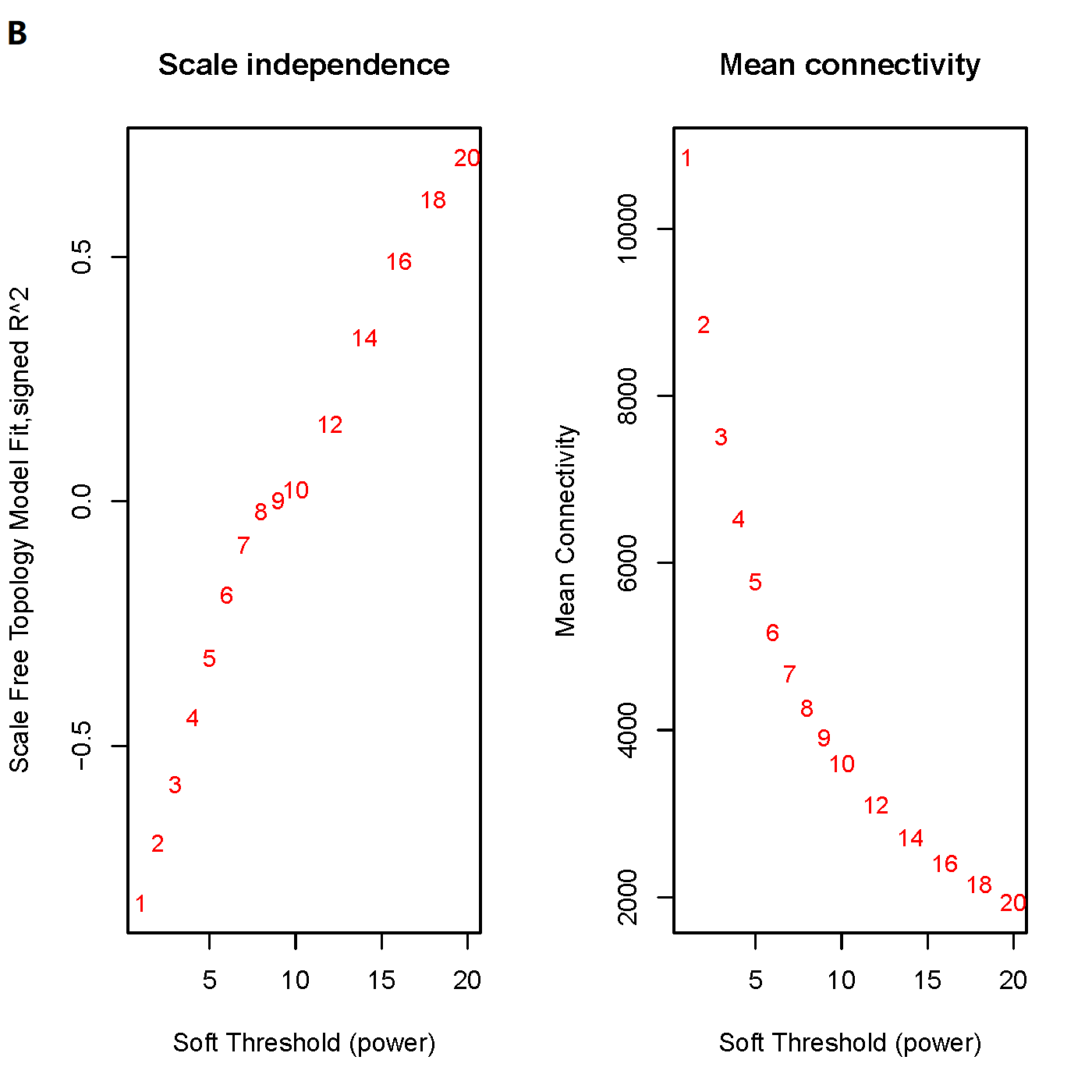

Supplement: Supplementary file 2 — Additional file 2: Figure S2. Detection of co-expressed modules based on weighted gene co-expression network analysis. (A) Clustering of the modules. (B) Analysis of network topology through different soft-threshold limits. [file 13020_2020_344_MOESM2_ESM.docx]
