# Supplementary material for: Transcriptome analyses revealed the ultraviolet B irradiation and phytohormone gibberellins coordinately promoted the accumulation of artemisinin in Artemisia annua L
Source: Chin Med. 2020 Jul 1;15:67. doi: 10.1186/s13020-020-00344-8 (PMC7329506; doi:10.1186/s13020-020-00344-8)

**Figure S1.** Functional annotation of all of genes into biological process, cellular component, and molecular function categories within the gene ontology (GO) database.(**A**) Gene ontology (GO) database of up-regulated genes; (**B**) Gene ontology (GO) database of down-regulated genes. (**C**) Number of differentially expressed genes detected between GA, UV and GA+UV treatments.


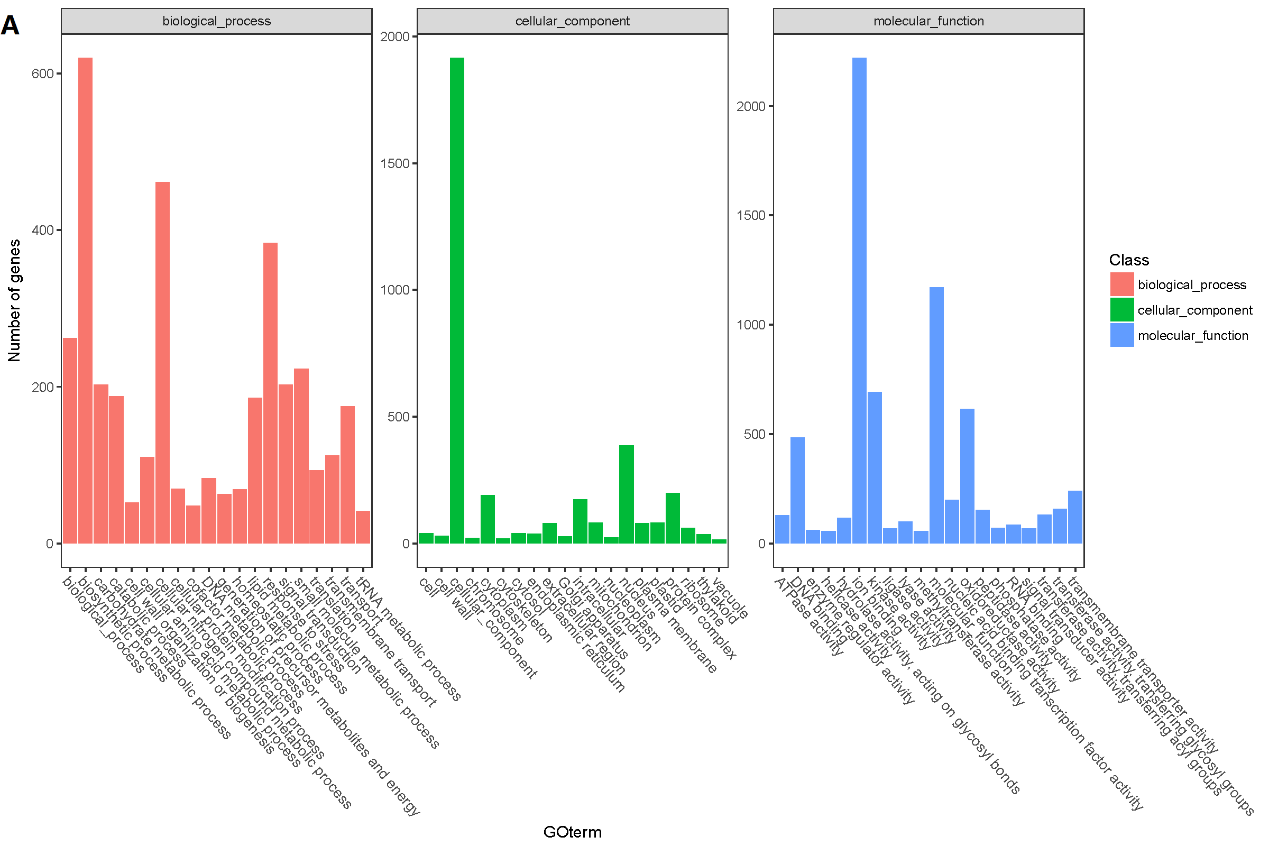


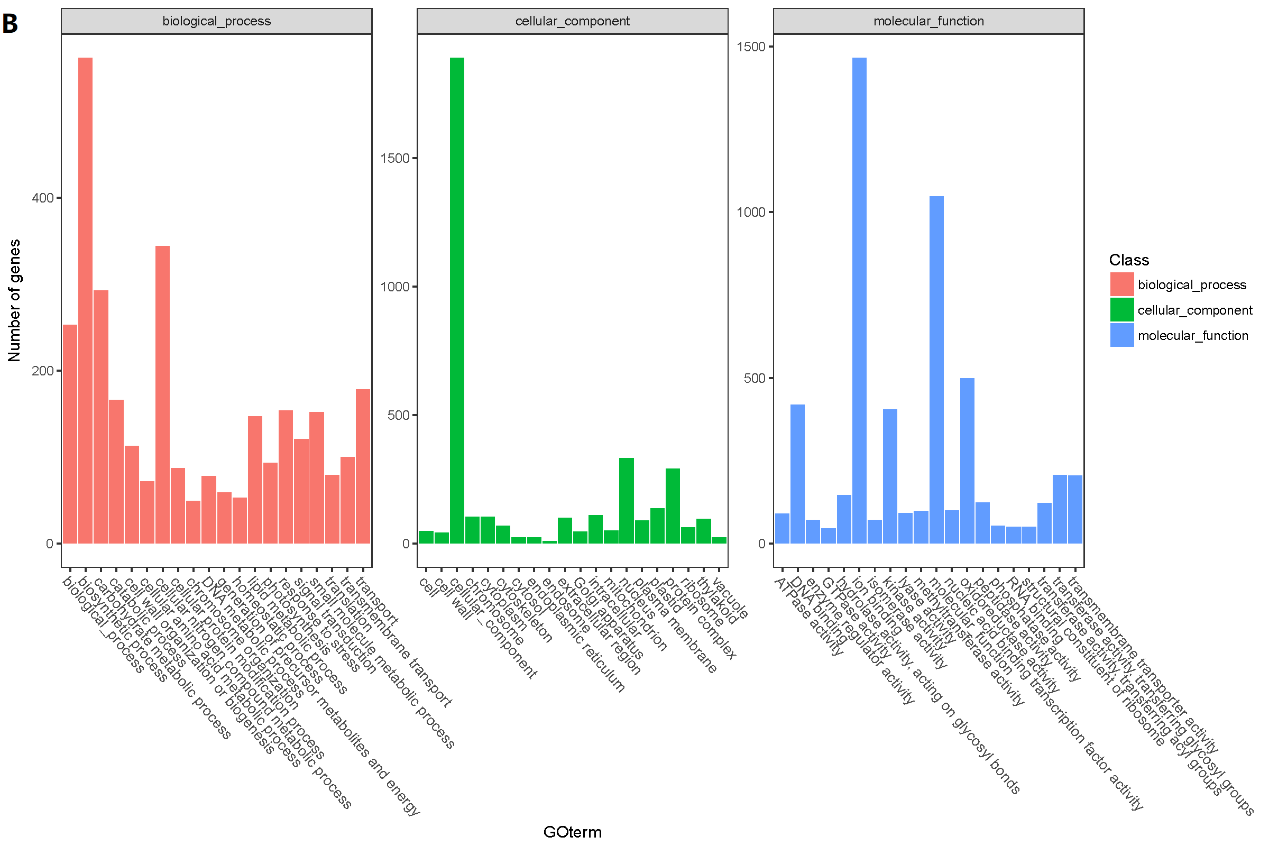


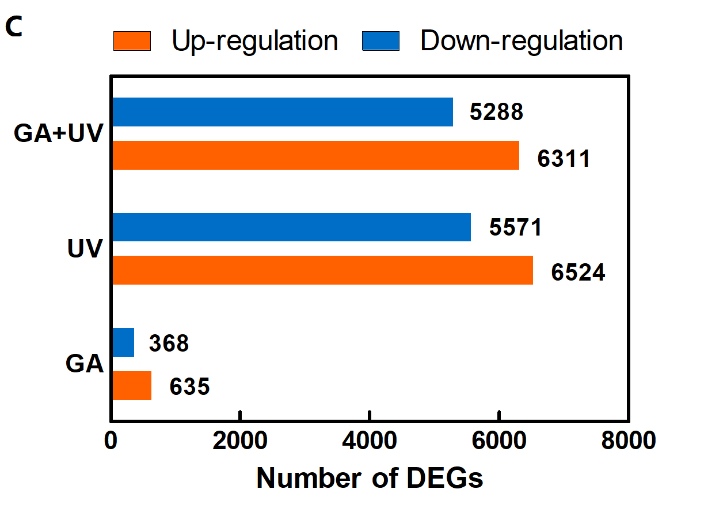

Supplement: Supplementary file 4 — Additional file 4: Figure S1. Functional annotation of all of the assembled unigenes into biological process, cellular component, and molecular function categories within the gene ontology (GO) database.(A) Gene ontology (GO) database of up-regulated genes; (B) Gene ontology (GO) database of down-regulated genes. KEGG pathway enrichment analysis of the annotated DEGs in the different treatments. (C) Number of differentially expressed genes detected between GA, UV and GA+UV treatments. [file 13020_2020_344_MOESM4_ESM.docx]
