# Supplementary material for: Transcriptome analyses revealed the ultraviolet B irradiation and phytohormone gibberellins coordinately promoted the accumulation of artemisinin in Artemisia annua L
Source: Chin Med. 2020 Jul 1;15:67. doi: 10.1186/s13020-020-00344-8 (PMC7329506; doi:10.1186/s13020-020-00344-8)
Supplement: Supplementary file 7 — Additional file 7: Figure S3. Weighted gene co-expression network analysis the co-expressed network of blue module (A), brown module (B) and yellow module (C), green module (D), turquoise module (E) and grey module (F). [file 13020_2020_344_MOESM7_ESM.docx]

**Figure S3.** Weighted gene co-expression network analysis the co-expressed network of blue module (A), brown module (B), yellow module (C), Green module (D), Turquoise module (E) and Grey module (F).


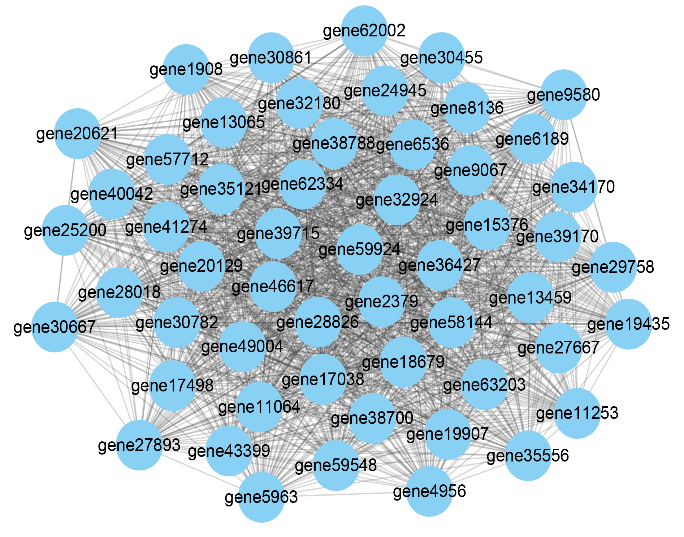


**A**


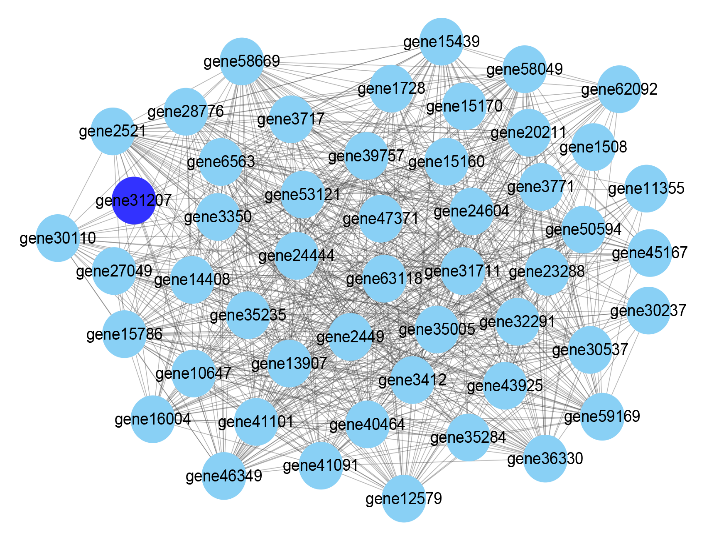


**B**


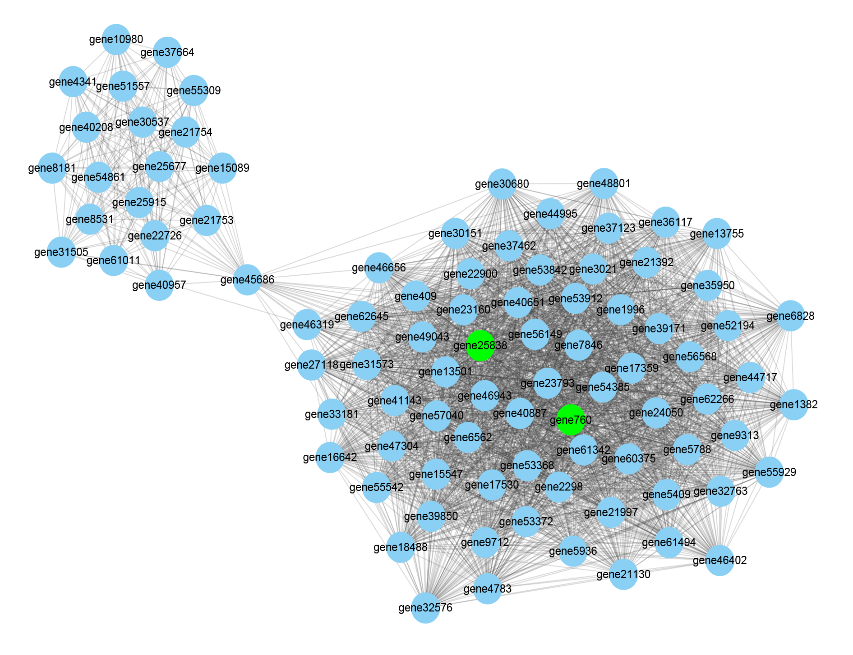


**C**


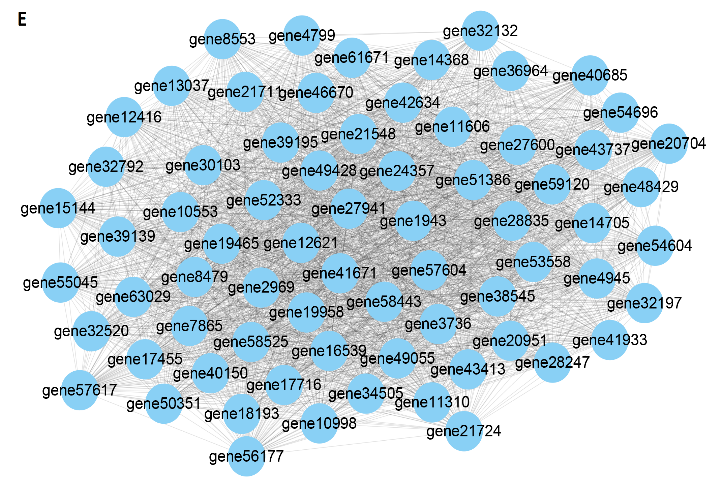


**E**


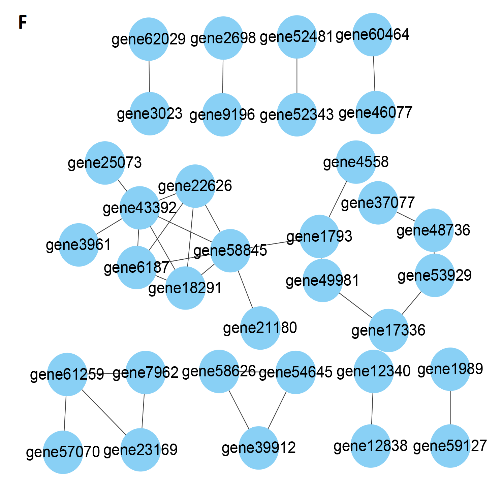

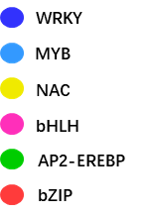


**F**


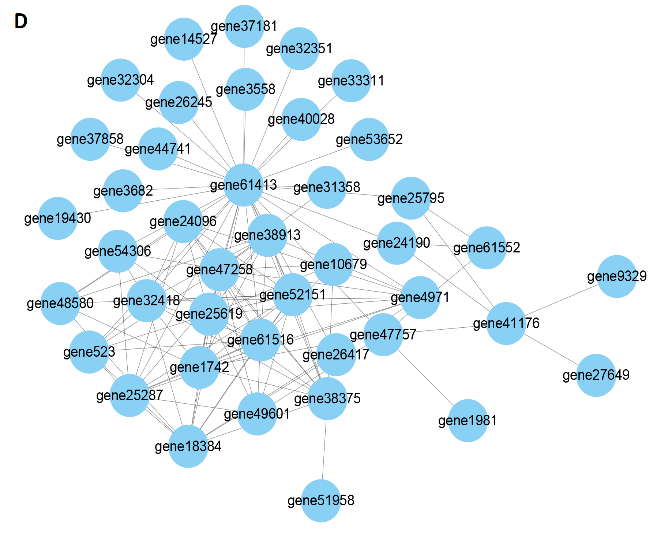


**D**
