# Supplementary material for: Transcriptome analyses revealed the ultraviolet B irradiation and phytohormone gibberellins coordinately promoted the accumulation of artemisinin in Artemisia annua L
Source: Chin Med. 2020 Jul 1;15:67. doi: 10.1186/s13020-020-00344-8 (PMC7329506; doi:10.1186/s13020-020-00344-8)

**Figure S4.** GO enrichment analysis of the co-expressed gene modules. (**A**) GO enrichment analysis of green module. (**B**) GO enrichment analysis of turquoise module. (**C**) GO enrichment analysis of grey module.


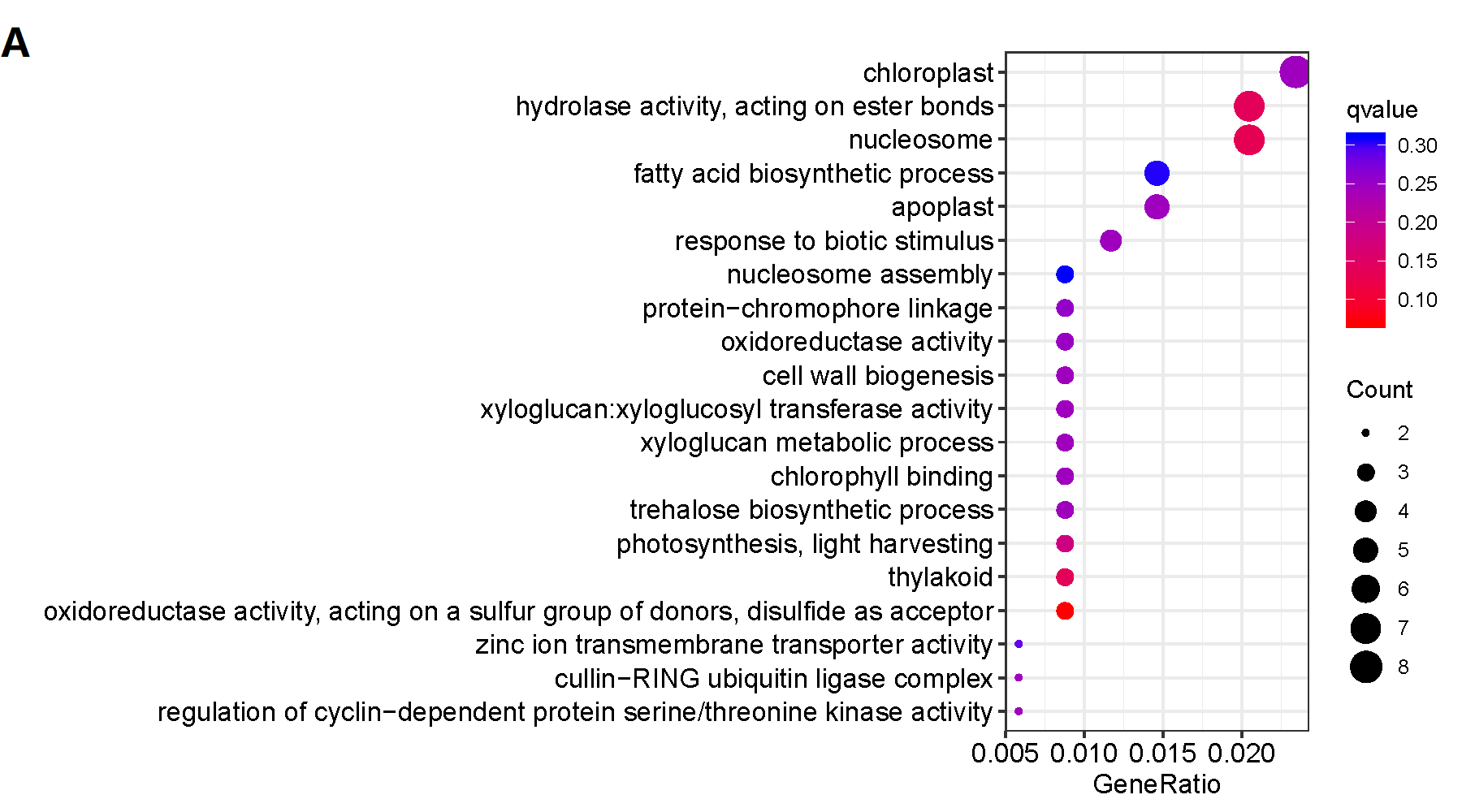


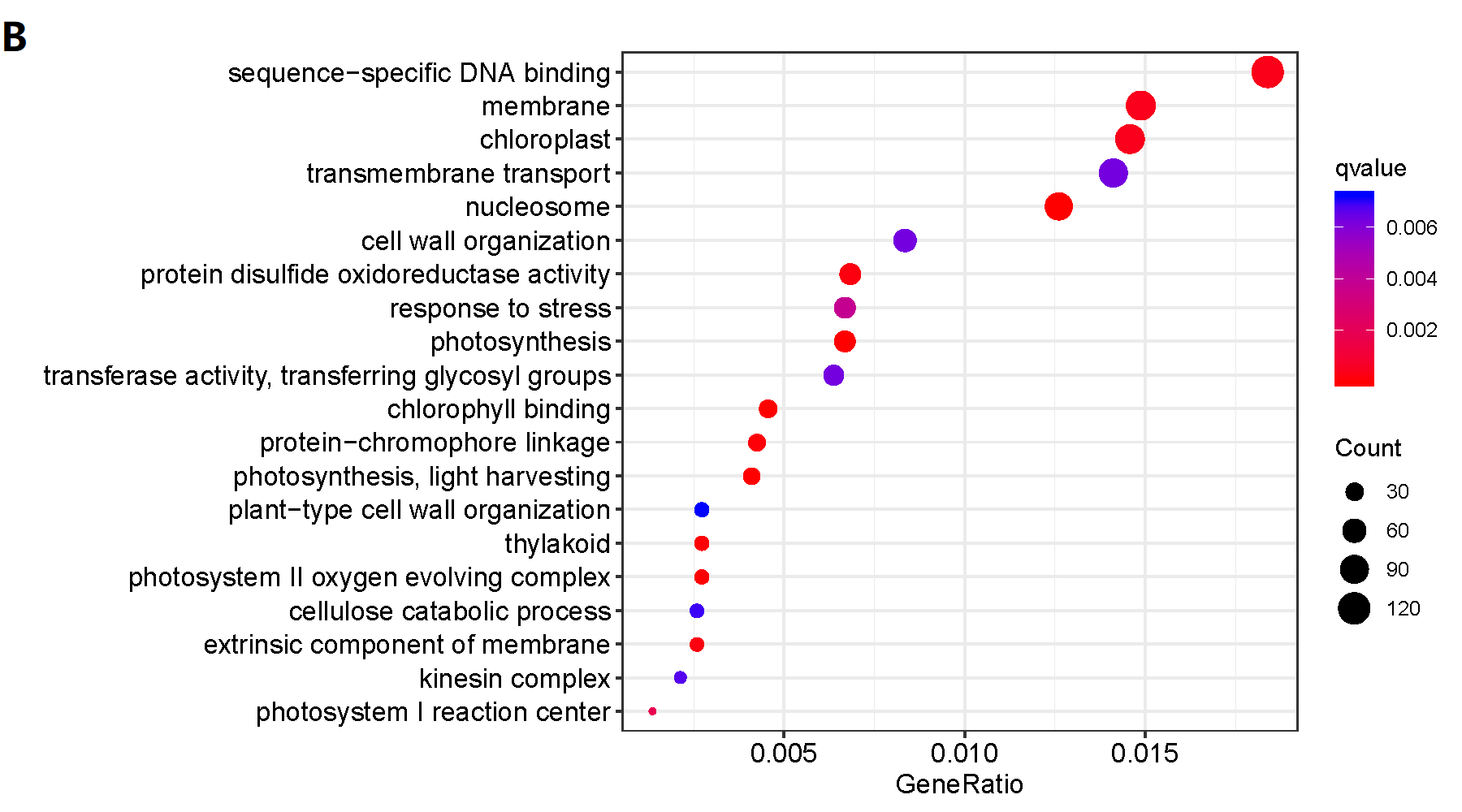


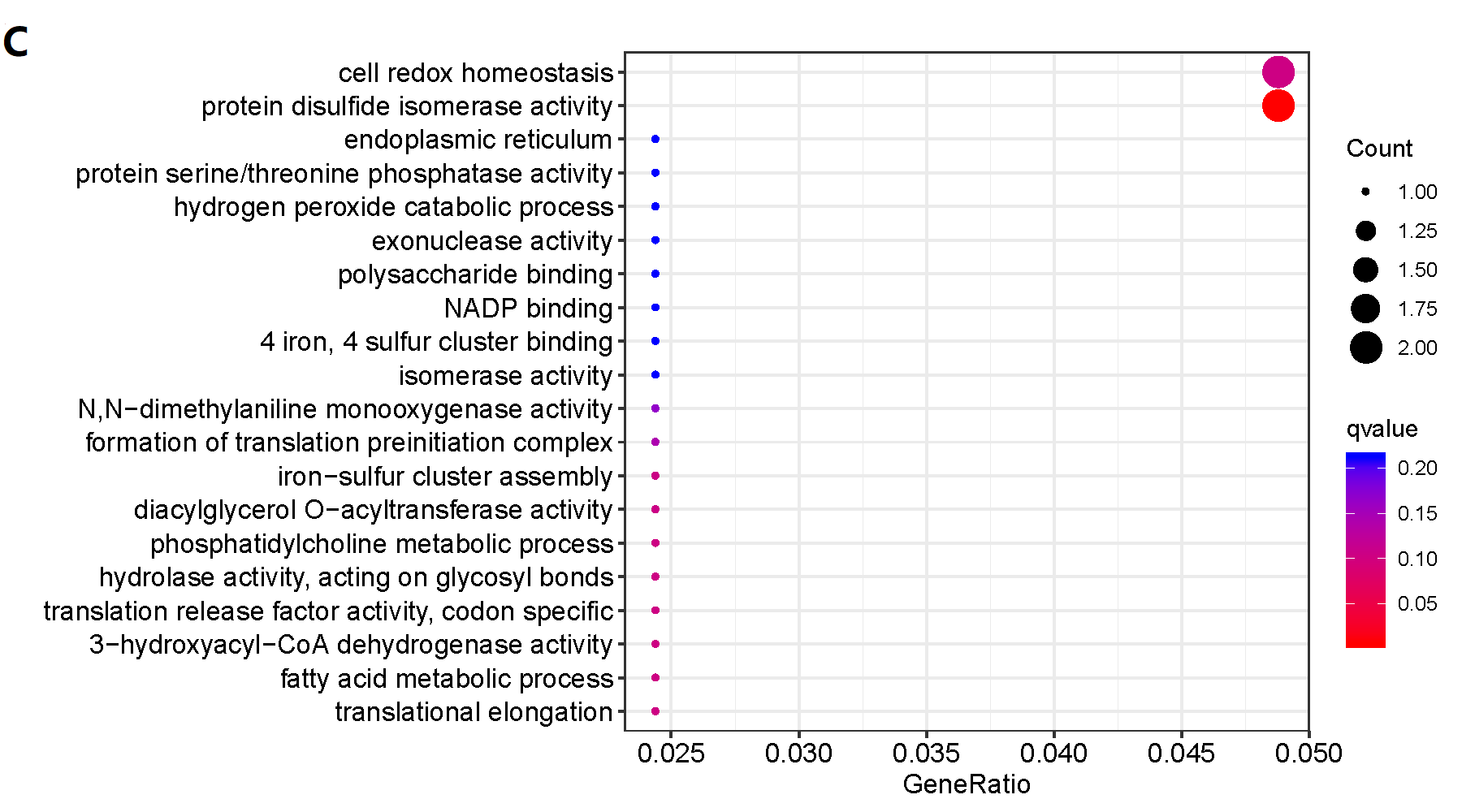

Supplement: Supplementary file 8 — Additional file 8: Figure S4. GO enrichment analysis of the co-expressed gene modules. (A) GO enrichment analysis of green module. (B) GO enrichment analysis of turquoise module. (C) GO enrichment analysis of grey module. [file 13020_2020_344_MOESM8_ESM.docx]
